# SUBSCAPULAR INJURY: PROSPECTIVE COMPARISON OF PHYSICAL EXAMINATION, MRI AND ARTHROSCOPY

**DOI:** 10.1590/1413-785220253301e285935

**Published:** 2025-02-03

**Authors:** Hélio Gonçalves Ribeiro, José Rodrigo Da Silva Ferreira, Flávio Wildon Dantas, Rodrigo De Araújo Santa Ritta

**Affiliations:** 1 Hospital Santa Casa de Misericórdia de Maceió, Department of Orthopedics and Traumatology, Maceió, AL, Brazil

**Keywords:** Rotator Cuff, Subscapularis, Arthroscopy, Rotator Cuff Injuries, Manguito Rotador, Músculo Subescapular, Artroscopia, Lesões do Manguito Rotador

## Abstract

Rotator cuff injury is the most frequent etiology of shoulder pain, with 24% of these injuries involving the subscapular tendon.

**Objective:**

To correlate the findings of three clinical tests (Gerber test, Belly Press test, and Bear Hug test) with Magnetic Resonance Imaging (MRI) and arthroscopic findings of subscapular lesions.

**Methods:**

Prospective cross-sectional study, from November 2023 to March 2024, with 50 patients with rotator cuff injury, evaluating sensitivity, specificity, and accuracy among clinical tests, MRI, and arthroscopic findings.

**Results:**

50 patients formed the sample, with 29 (58%) men and 21 (42%) women aged 42 to 86 years. We found a specificity of 88% and an accuracy of 54% in MRI. Regarding the Gerber test, the Belly Press test, and the Bear Hug test, the sensitivity was 64%, 64%, and 76%, with specificity of 75% for the Gerber and Belly Press tests and accuracy of 74% for the Bear Hug test.

**Conclusion:**

We concluded that the Bear Hug test showed higher sensitivity and accuracy in detecting subscapular tendon lesions, with MRI being the most specific method. ***Level of Evidence II; Prospective Study.***

## Resumo

A lesão do manguito rotador é a etiologia mais frequente de dor no ombro e 24% destas lesões podem envolver o tendão subescapular.

**Objetivo:**

Correlacionar os achados de três testes clínicos (teste de Gerber, Belly Press test e Bear Hug test) com imagens de Ressonância Nuclear Magnética (RNM) e achados artroscópicos das lesões do subescapular.

**Métodos:**

Estudo transversal prospectivo, no período de novembro de 2023 a março de 2024, com 50 pacientes portadores de lesão do manguito rotador, avaliando a sensibilidade, especificidade e acurácia entre os testes clínicos, RNM e achados artroscópicos. Resultados: 50 pacientes formaram a amostra, sendo 29 (58%) homens e 21 (42%) mulheres, com idade variando de 42 a 86 anos. Encontramos uma especificidade de 88% e acurácia de 54% na RNM. Com relação aos testes de Gerber, Belly Press test e Bear Hug, a sensibilidade foi, respectivamente, de 64%, 64% e 76%, sendo a especificidade de 75% para o teste de Gerber e Belly Press test, e acurácia de 74% para o Bear Hug test.

**Conclusão:**

Concluímos que o Bear Hug test apresentou maior sensibilidade e acurácia na detecção de lesões do tendão subescapular, sendo a RNM o método mais específico. **Nível de Evidência II; Estudo Prospectivo.**

## INTRODUCTION

Rotator cuff injury is the most frequent etiology of shoulder pain with an incidence of 14.7 per 1000 patients. Although the supraspinatus tendon is the most commonly affected of the cuff muscles, approximately 24% of these injuries may involve the subscapularis tendon.^1^,^2^ Some authors propose that 37% of these pathologies involve the subscapularis; however, it is still poorly recognized, underdiagnosed and forgotten.^3^ The subscapularis is the strongest and widest muscle of the rotator cuff, allowing internal rotation of the humerus, providing anterior stability to the shoulder and being involved in the force balance of the glenohumeral joint.^4^ Being responsible for 50% of the total force of the rotator cuff, its integrity is a prerequisite for a variety of reconstructive techniques in the rotator cuff and its injury leads to pain, functional disability, and shoulder instability.^5^

A variety of clinical signs and diagnostic tests have been published to assess the integrity of the subscapularis,^6^ among them we highlight the Lift-off test (Gerber test) described by Gerber and Krushell et al.,^7^ the Belly Press test also described by Gerber et al.,^7^ and the Bear Hug test described by Barth et al.^8^ Despite a variety of tests, subscapularis injury continues to be undiagnosed in clinical practice, probably because of the different diagnostic values of each test used alone, with no consensus establishing which is the best clinical test with good sensitivity and specificity. None of these maneuvers, however, present satisfactory sensitivity and specificity, resulting in low positive predictive values.

MRI is considered an important tool in the diagnosis of subscapularis injuries, but its accuracy is lower than for detecting other rotator cuff injuries.^3^,^9^,^10^ Despite advances in MRI technique and equipment, there is still difficulty in diagnosing subscapularis injuries, with sensitivity ranging from 25-94% and specificity from 64-100%.^11^ In the study by Smith et al.,^12^ sensitivity and specificity of 80% and 95%, respectively, were found for partial rotator cuff tears, and 91% and 97%, respectively, for complete rotator cuff tears.

With the advent of arthroscopy in shoulder surgery, the understanding of pathologies has been improving. Although considered the gold standard for the diagnosis of these injuries, there are limitations in the evaluation of the subscapularis tendon, especially in its inferior portion, often requiring the use of specific intraoperative maneuvers or even optics with expanded vision, such as 70° optics, which frequently are not routinely available.^3^,^13^ In successive arthroscopies, Barth et al. observed that several lesions of the upper portion of the subscapularis tendon were not predicted by the Belly Press test and Lift-off test maneuvers, since the uppermost fibers were recruited only in internal rotation of the shoulder with the elbow in a more anterior position.^8^

The aim of this study is to correlate the findings of three clinical tests with MRI images and arthroscopic findings of subscapularis injuries, determining the diagnostic value of these tests.

## MATERIALS AND METHODS

A prospective cross-sectional study was conducted from November 2023 to March 2024, evaluating the sensitivity, specificity, accuracy, positive predictive value and negative predictive value of the correlation of three clinical tests (Lift-Off test or Gerber test, Belly Press test or Abdominal Press Test, and Bear Hug test) for the diagnosis of subscapularis tendon injury with MRI images and intraoperative arthroscopic findings. Sixty patients of both sexes, over 18 years old, diagnosed with rotator cuff injury and requiring surgical intervention, were evaluated. All clinical tests and arthroscopies were performed by the same surgeon with over 15 years of experience in shoulder and elbow surgery. Our project was submitted to the Research Ethics Committee under CAAE: 71053723.9.0000.0155. Informed consent forms were obtained from all patients following Resolution 466/12 of the National Commission for Research Ethics.

The following exclusion criteria were used: previous shoulder surgeries, polytraumatized patients, alcohol or illicit drug abuse, open injuries, neoplastic diseases, associated upper limb fractures, adhesive capsulitis, glenohumeral arthrosis, psychiatric illness, pregnancy, clinically uncompensated comorbidities, and active infection. Ten patients were not eligible for the study: 6 due to glenohumeral arthritis, 3 with a diagnosis of adhesive capsulitis, and 1 with schizophrenia.

Epidemiological data such as age, gender, and laterality were collected at the initial consultation and a preoperative clinical evaluation was performed in the outpatient clinic using the three clinical tests of Gerber, Bear Hug test, and Belly Press test. Regarding MRI evaluation, only exams performed in the last 6 months before arthroscopy were considered.

For MRI, the patient was placed in a supine position with arms in the neutral position. All MRIs were performed with a 1.5 Tesla resolution using a shoulder support. The imaging protocol included T2-weighted coronal oblique, oblique sagittal, axial with fat suppression, and T1-weighted coronal oblique and oblique sagittal sequences.

In the surgical suite, a new clinical evaluation using the three clinical tests and reevaluation of MRI were performed, followed by arthroscopic technique to confirm or rule out the presence of subscapularis injury. All patients were operated on in a beach chair position under general anesthesia and brachial plexus block. Conventional arthroscopic portals (posterior, anterior, lateral) were used. Complete joint exploration of the glenohumeral joint and subacromial space through the posterior portal using 30° and 70° optics along with the 30°-40° flexion and internal rotation maneuver to assess subscapularis injuries associated with anterior portal probing were performed. The subscapularis was evaluated and classified according to Lafosse, who subdivides it into 5 types of lesions where type 1 refers to partial tear of the upper 1/3, type 2 to complete tear of the upper 1/3, type 3 to complete tear of the upper 2/3 of the tendon, type 4 refers to a complete rupture, and type 5 to a complete irreparable rupture with static anterosuperior subluxation.^14^ Statistical analysis of the patients was performed using descriptive statistics and analyzed using the Jamovi module. The number of true positives, true negatives, false positives and false negatives were used to determine sensitivity, specificity, negative predictive value, positive predictive value and accuracy of the clinical tests and MRI with a 95% confidence interval using arthroscopic findings as the gold standard.^15^

## RESULTS

The sample consisted of 50 patients, with 29 (58%) males and 21 (42%) females, ranging in age from 42 to 86 years (mean = 60.53; SD = 9.61), with the right side being the most affected with 37 cases (74%). Of the 42 lesions confirmed by arthroscopy, 24 were classified as Lafosse type 1 (57.1%) and 17 (40.4%) were classified as Lafosse type 2 and 3 (Table 1).

**Table 1:** Epidemiological and clinical characteristics (n = 50).

Characteristic		M (±SD) or n (%)	
Age, years, M(SD)		60,53 (± 9,61)	
Gender	Male, n (%)	29	(58)
	Female, n (%)	21	(42)
Laterality	Right, n (%)	37	(74)
	Left, n (%)	13	(26)
Lafosse	1 - n (%)	24	(57,1%)
	2 and 3- n (%)	17	(40,4%)
	4 – n (%)	1	(2,3%)

Source: developed by the author, 2024.

### Magnetic Resonance Imaging

The reported results are from a medical decision test applied to 50 individuals, of which 42 were identified as diseased (Gold Positive) and 8 as healthy (Gold Negative) by the gold standard (arthroscopy), which is the reference method for diagnosis (Table 2).

**Table 2:** Arthroscopic confirmation vs. MRI diagnosis.

Diagnosis confirmation	Nº of cases	N (%)
Diseased	42	84
Healthy	8	16
Positive Tests	21	42
Negative Tests	29	58
True Test	27	54
Wrong Test	23	46

Source: developed by the author, 2024.

The imaging test in question demonstrated lower sensitivity with a value of 48%, compared to the three clinical tests; however, it presented higher specificity with a value of 88%, indicating that of the 8 individuals considered healthy by the gold standard, 7 were correctly identified as healthy by the test (Test Negative). Furthermore, it presented a positive predictive value (PPV) of 95%, being higher among all, indicating that the probability of an individual with a positive result on the test actually having the disease is 95%. In contrast, it showed lower negative predictive value (NPV) and accuracy, being 24% and 54%, respectively (Table 2 and 3).

**Table 3:** Sensitivity, specificity, accuracy, positive predictive value (PPV) and negative predictive value (NPV) by diagnostic technique.

	MRI	Gerber Test	Belly Press Test	Bear Hug Test
Sensitivity	48%	64%	64%	76%
Specificity	88%	75%	75%	63%
Accuracy	54%	56%	66%	74%
Positive Predictive Value (PPV)	95%	93%	93%	91%
Negative Predictive Value (NPV)	24%	29%	29%	33%

Source: developed by the author, 2024.

Additionally, the positive likelihood ratio was 3.81, indicating that a positive result on the test is 3.81 times more likely in diseased individuals than in healthy individuals. The 95% confidence intervals mean that, with 95% certainty, the true sensitivity of the test is between 32% and 64%, and the true specificity is between 47% and 100%. The diagnostic odds ratio was 6.36, with a 95% confidence interval ranging from 0.72 to 56.35 (Table 4).

**Table 4:** Odds ratio correlation between clinical tests and MRI.

		95% confidence interval		
	Statistical decision	Estimate	Lower	Higher
MRI	Odds ratio	6.36	0.72	56.35
Gerber test	Odds ratio	5.40	0.97	30.16
Belly press test	Odds ratio	5.40	0.97	30.16
Bear hug test	Odds ratio	5.33	1.08	26.36

Source: developed by the author, 2024.

### Gerber test

The test in question demonstrated sensitivity, specificity, PPV, and NPV values of 64%, 75%, 93%, and 29%, respectively, being similar to the values found in the Belly Press test; however, it presented lower accuracy (56%). The 95% confidence intervals for the sensitivity and specificity of the test were from 48% to 78% and from 35% to 97%, respectively (Table 3).

### Belly press test

As described above, it presented some similar data to the Gerber test but with better accuracy, at 66%, showing that it had the best proportion of correct results compared to the Gerber test. Furthermore, it presented a diagnostic odds ratio value of 5.40, with a 95% confidence interval from 0.97 to 30.16 (Table 3 and 4).

### Bear hug test

Of the 42 diseased individuals, the test correctly identified 32 as diseased (Test Positive), resulting in a sensitivity of 76%, which was higher than that found in the other clinical tests and even in MRI. This indicates that the test has a 76% chance of correctly identifying a diseased individual, with a 95% confidence interval for sensitivity between 61% and 88%. It also presented the highest accuracy, with a value of 74%, favoring having the highest proportion of correct results (true positives and true negatives) in relation to the total tests performed, compared to the other clinical tests and MRI. Additionally, it presented an NPV of 33%, higher than other clinical tests and MRI, indicating a higher probability of an individual with a negative test actually not having the disease (Table 2 and 3).

## DISCUSSION

Over the years, several studies have shown that subscapularis tendon injuries are not just occasional occurrences; a consistent increase in rates has led to an increase in its prevalence.^16^,^17^

Bennet et al.^16^ demonstrated a prevalence of 27%, Bartsch et al.^6^ 30%, and Barth et al.^8^ 58.8% of subscapularis injuries in their studies. Our study showed a prevalence of 84%, much higher than most studies in the literature, with only patients undergoing surgical treatment for rotator cuff injuries being evaluated here. The advent of arthroscopy with the use of 70° optics, cameras with 4k definition in modern devices, LED fiber optics, in addition to the described maneuvers for intraoperative use that facilitate visualization of the subscapularis insertion on the lesser tuberosity, explains the increased diagnosis of subscapularis injuries, especially partial lesions that are more difficult to detect on MRI due to the absence of indirect signs, resulting in an increased prevalence of these injuries. When evaluating the accuracy of MRI in detecting subscapularis injuries, we found in our study an accuracy value of 54% with an NPV of 24%, which is contrary to that described by Adams et al.^9^ in their study, with an accuracy of 82% and NPV of 78%. Pfirrmann et al.^18^ evaluated the result of two musculoskeletal radiologists in predicting subscapularis injury, assessing MRI and their interpretations, which were compared with intraoperative findings; regarding sensitivity, it was reported 91% for both the first and second radiologists, showing a difference compared to our study, which showed a value of 48%. Regarding specificity, the first was 86%, and the second was 79%, which is consistent with our research. Malavolta et al.^19^ found in their systematic review an accuracy of 90%, with sensitivity of 68% and specificity of 90%, similar to the 88% found in our study, making it the most specific method in our results.

In this study, most of the lesions found in arthroscopy that were not detected in MRI images by radiologists were partial, whether articular or intrasubstance. Although they were made with the same protocol, MRI images were taken and evaluated in 3 different locations by different radiologists. The method has a low capacity to diagnose subscapularis injury when compared to other rotator cuff tendons, requiring greater attention from radiologists in their evaluations and the use of other imaging protocols to develop clearer signs of subscapularis tendon injury. Additionally, we emphasize that the improvement in technology for performing arthroscopies has facilitated intraoperative diagnosis.^19^

Regarding the clinical tests evaluated in this study, we observed greater sensitivity with the Bear Hug test, totaling 76%, a result also observed by Schiefer et al.^20^. Barth et al.^8^ in their work describing the maneuver found a sensitivity of 60%, suggesting that the test is especially useful in detecting lesions of the upper fibers of the subscapularis. This is supported in this study when we evaluate Table 1 and find the majority of cases to be type 1 and 2, according to Lafosse’s classification. The sensitivity of the Gerber test (Lift-off test) was 64% in our study, differing from several other studies found in the literature such as that of Bartsch et al.^6^ with 40%, Schiefer et al.^20^ with 25%, and Kappe et al.^5^ with 35%. The sensitivity of the Belly Press test, which was also 64%, shows a similar percentage to what we found in the literature, such as in Barth et al.^8^, with 76%. Perhaps an explanation for the discrepancy in the results found regarding the Gerber test is due to the various modifications in its execution and interpretations.

Although this study shows greater sensitivity with the Bear Hug test, especially for lesions of the upper 1/3, the sensitivity of clinical tests proved to be limited, emphazising the importance of performing all tests along with the clinical history to detect the greatest number of lesions, hereby ensuring that this diagnosis does not go overlooked. It is also important for radiologists to develop a more detailed protocol using axial and sagittal cuts of MRI to increase accuracy in diagnosing subscapularis injuries.

Our study has some limitations, such as the small sample presented, the fact that the majority of diagnosed lesions were partial and the variability in the assessment of MRI images, considering they were not conducted in a single center.

## CONCLUSION

We conclude that the findings of our study show that the Bear Hug test was the physical examination maneuver that presented the highest sensitivity and accuracy in detecting subscapularis tendon injuries, with MRI being the most specific method.
